# On the importance for drug discovery of a transnational Latin American database of natural compound structures

**DOI:** 10.3389/fphar.2023.1207559

**Published:** 2023-06-22

**Authors:** Timothy M. Thomson

**Affiliations:** ^1^ Institute for Molecular Biology (IBMB-CSIC), Barcelona, Spain; ^2^ CIBER de Enfermedades Hepáticas y Digestivas (CIBERehd), Madrid, Spain; ^3^ Universidad Peruana Cayetano Heredia, Lima, Peru

**Keywords:** natural products, virtual screening, Latin America, databases, computation

## Introduction

Drug development is a complex, risky, expensive and time-consuming process that requires the accurate execution of multiple stages, from identification and selection of collections of potentially druggable molecules to proof-of-concept validation. Prior to embarking on a drug discovery project, a detailed strategic plan must be designed that includes hundreds of critical considerations such as source of compounds for screening, feasibility of their synthetic pathways, target selection (molecules, cells, organisms), type of output (binding, function, phenotype), throughput, nature, layers and iterations of the screening process, scoring systems, lead optimization approaches or model systems for validation (biochemical activity, cellular function, organismal properties) and, crucially, good contingency plans.

The need to discover new drugs rests on practical matters of human progress, rather than mere market considerations. For example, the global emergence of multidrug resistant pathogens makes it a research target to find alternative antibiotics; current cancer drugs, including advanced biologicals, face drug resistance; many parasitic diseases lack effective drug treatments; highly prevalent neurodegenerative diseases and rare diseases, which collectively affect significant numbers of people, are essentially drug orphan; vital agricultural crops are plagued by fungal and parasitic diseases that have evolved to become increasingly resistant to currently available chemicals. The current COVID-19 pandemic illustrates how pharmaceutically unprepared humanity is (vaccines aside) to confront the sudden emergence of a novel, deadly and highly transmissible pathogen. To date, only a handful of repurposed drugs display demonstrable therapeutic efficacy against infection and disease by the causing agent, SARS-CoV-2 (Lui and Guaraldi, 2023; Sandulescu et al., 2023). Despite an unprecedented parallel effort by hundreds of thousands of industrial and academic scientists worldwide, employing leading-edge technologies, no new drugs have been discovered over the past 3 years to effectively treat this disease.

## Natural products as sources of chemical diversity

That some of these targets and diseases may be truly undruggable remains a possibility. However, the general working hypothesis is that small molecules or biologicals will be eventually found to match the majority of designated new targets and to significantly improve upon existing drugs that act on more conventional targets. Although this tenet may seem like wishful thinking, it is at least partly based on sound estimates of ligand structural diversity and druggable chemical space, as well as on the evidence that combinatorial approaches remain largely untested. As such, while the largest currently available compound and fragment libraries, used in ultra-large virtual screenings, contain up to 2 × 10^9^ unique structures (Lyu et al., 2019; Grygorenko et al., 2020; Crunkhorn, 2022), the total chemical space of small organic compounds suitable for drug discovery is estimated as more than 10^60^ molecules (Bohacek et al., 1996). The underlying concept is that the more compounds are screened, the higher the likelihood of finding true positives (Lyu et al., 2019; Gorgulla et al., 2020).

The above considerations suggest that there is still ample margin to finding new structures to be used as ligands for drug discovery screening efforts, which begs the question: Where will new chemical entities (NCEs) likely come from? Because of their special features as compared to currently available synthetic molecules, natural products (NPs) offer advantages as sources of future NCEs. As such, NPs provide large scaffold diversity and structural complexity accompanied with generally higher molecular rigidity, more chiral centers, higher fraction of sp^3^ atoms, more oxygen atoms and hydrogen bond acceptors and donors, lower octanol–water partition coefficients (cLog) indicating higher hydrophilicity, low ratio of aromatic ring atoms or diversity of ring systems (Koehn and Carter, 2005; Atanasov et al., 2021; Najmi et al., 2022).

A major limitation for expanding the ligand chemical space with NPs is the laborious nature of NP isolation and structural characterization towards drug discovery. Traditionally, this is done through producing crude extracts with a variety of solvents, screened and fractionated guided by biological activity, and hit compounds purified and structurally characterized. Given the availability of large compound structural databases, a virtual screening-centric strategy may afford to reverse conventional drug discovery schemes. As such, compounds can be structurally characterized after minimal purification or fractionation from crude extracts, by means of NMR spectroscopy, high-resolution mass spectrometry (HRMS), liquid chromatography HRMS (LC–HRMS) (Giavalisco et al., 2008; Wolfender et al., 2019; Garcia-Perez et al., 2020; Stavrianidi, 2020). These methods enable routine acquisition of accurate molecular mass information and unambiguous assignment of formulae for hundreds to thousands of metabolites in a single extract over a broad dynamic range (Fontana et al., 2020), thus facilitating chemical entity dereplication (Arora and Banerjee, 2019). In turn, dereplication is aided by accessing databases such as the Dictionary of Natural Products (https://dnp.chemnetbase.com/), which encompasses all NP structures reported with links to their biological sources, the Global Natural Products Social (GNPS) molecular networking platform (https://gnps.ucsd.edu/) (Wang M. et al., 2016), in which thousands of sets of MS/MS data are recorded from a given set of extracts, clustering compounds by their structural relationships (Allard et al., 2016; Zhou et al., 2017; da Silva et al., 2018), Compound Structure Identification (CSI) (Aksenov et al., 2017) or METLIN (Guijas et al., 2018), containing fragment ion spectra that can be used for the identification of unknown compounds. In summary, quantitative NMR and LC–MS approaches can yield novel structures to populate screening-ready databases at early stages in virtual screening drug discovery strategies, thus avoiding futile downstream development efforts (Wohlgemuth et al., 2016).

In spite of these technological advances that facilitate the expansion of the known chemical space, NPs may contain only a fraction of the theoretical space and scaffold diversity (Pye et al., 2017). In order to further expand chemical space and structural diversity, several strategies have been used to create new biologically active compounds by adding appendages on NP core scaffolds (Grigalunas et al., 2022), such as diversity-oriented synthesis (DOS) (Schreiber, 2009), DNA encoded libraries (DEL) (Franzini and Randolph, 2016) or biology-oriented synthesis (BOS) (van Hattum and Waldmann, 2014). Other strategies go beyond the available NP scaffolds by resorting to ring distortion reactions (Motika and Hergenrother, 2020), albeit still relying on the original scaffolds. The pseudo-NP strategy deconstructs NPs into fragments and recombines them into novel scaffolds that are not possible to attain through known biosynthetic pathways but retain the chemical and biological relevance of NPs (Grigalunas et al., 2020; Karageorgis et al., 2020).

Additional efforts to expand the NP chemical space include engineering biosynthetic pathways aimed at yielding new NP analogues with potentially improved pharmacological properties (Atanasov et al., 2021). Such strategies include the activation of cryptic or occult biosynthetic gene clusters that remain otherwise silent (Macheleidt et al., 2016), which can be achieved through the manipulation of culture conditions (Pan et al., 2019), micro-organism co-cultures (Bertrand et al., 2014) or exposure to small molecule epigenetic modulators (Pillay et al., 2022), among other approaches. The expansion of chemical space through various strategies entails the parallel development of new chemical methods capable of solving previously untested synthetic paths, so as to produce compounds corresponding to the newly designed structures and in cost-effective yields (Cai et al., 2023).

Further to approaching theoretical ligand chemical space limits and structural characterization of NP and NP-like molecules, a major challenge is to make them available as large screening-ready libraries. Several databases provide information on NPs and their structures (Table 1). Compared to these databases, the currently accessible databases of chemical entities with focus on NPs of Latin American origin contain information on relatively few compounds (reviewed in (Medina-Franco, 2020; Nunez et al., 2021; Gomez-Garcia and Medina-Franco, 2022)) (Table 2). As such, given the estimated share of Latin American biodiversity in global biodiversity (Raven et al., 2020), it is apparent that NPs of Latin American origin are heavily underrepresented in databases of NP physicochemical properties and structures.

**TABLE 1:** Natural Product databases containing NP structural information.

Database	URL/References	Number of NPs
PubChem	https://pubchem.ncbi.nlm.nih.gov/(Wang et al., 2009)	1 × 10^8^ (synthetic and NP)
SuperNatural	https://bioinf-applied.charite.de/supernatural_3/Dunkel et al. (2006); Banerjee et al. (2015); Gallo et al. (2023)	4.5 × 10^5^
COCONUT	https://coconut.naturalproducts.net/ Sorokina et al. (2021)	4.0 × 10^5^
Dictionary of Natural Products	https://dnp.chemnetbase.com/	3 × 10^5^
ChEMBL	https://www.ebi.ac.uk/chembl/(Gaulton et al., 2017)	2.4 × 10^6^
Natural Products Atlas	https://www.npatlas.org/ Wang et al. (2016a)	2.4 × 10^5^
NAPRALERT	https://napralert.org/	3.0 × 10^5^
MarinLit	http://pubs.rsc.org/marinlit/) Blunt et al. (2018)	2.7 × 10^4^
TCM Database@Taiwan	https://tcm.cmu.edu.tw/ Chen (2011)	6.4 × 10^4^

**TABLE 2:** Databases of chemical entities with focus on NPs of Latin American origin.

Database	URL/References	Number of NPs
LNMol	http://lnmol.iq.usp.br/	>5,000
ChEMBL-NTD	https://www.ebi.ac.uk/chemblntd/	>10,000
NuBBE DB	http://nubbe.iq.unesp.br/	>1,500
BRENDA NPAtlas	https://www.brenda-enzymes.org/npatlas/index.php	>3,000
UEFS	http://zinc12.docking.org/catalogs/uefsnp	503
BIOFACQUIM	Sánchez-Cruz et al. (2020)	553
CIFLORPAN	Olmedo et al. (2017)	450

Large chemical structure databases are necessary for next-generation virtual drug discovery efforts, but they are not sufficient. Open-source, robust platforms are also needed that can integrate tasks in virtual screening and provide smooth connectivity to docking tools, such as VirtualFlow (Gorgulla et al., 2020), which can dock 1 billion compounds in about 2 weeks when run on 10,000 CPU cores, or V-SYNTHES (Sadybekov et al., 2022), which performs iterative steps of library preparation, enumeration, docking and hit selection, handling fragment-like libraries representing all possible scaffold–synthon combinations for all reactions in the 11 billion compound REAL Space library.

## Novel approaches to unbiased high-throughput target identification

Experimental approaches for target identification require molecular and biochemical studies of disease pathophysiology (McFedries et al., 2013; Shaker et al., 2021; Zecha et al., 2023), which can be costly, labor-intensive and time-consuming. Conventional virtual screening approaches for target selection focus on a preferred molecular target to conduct structure-directed screenings. For better outcomes, such targets need to be structurally resolved at the highest possible atomic resolution. Until recently, that entailed “one target at a time” strategies. For decades, conventional approaches to the resolution of macromolecular structures have relied on techniques such X-ray crystallography or NMR, which are labor-intensive and low-throughput. The advent to fruition of cryoelectron microscopy (Baumeister, 2022) has enormously speeded up this process. As a result of these collective efforts, there are currently over 200,000 experimentally resolved structures deposited in Protein Data Bank (https://www.rcsb.org/), as unique entries corresponding to full-length proteins, fragments and complexes (protein-protein, protein-DNA, protein-ligand). This volume of structural information has laid the foundation for, and enabled, the use of machine learning tools, such as AlphaFold2 (Jumper et al., 2021) or RoseTTA fold (Baek et al., 2021), to accurately predict the structures of millions of proteins. As such, the AlphaFold protein structure database (https://alphafold.ebi.ac.uk) currently contains 214,683,829 predicted structures, including 48 complete proteomes. An added bonus to these predictive tools is that targets for which the experimentally determined structures are incomplete or ambiguous at specific regions can be completed or “polished” for subsequent use in virtual screening. For virtual drug discovery, potential binding sites must be defined on target proteins. To this end, a number of tools have been developed to predict pockets amenable to blocking by small drug-like molecules on proteins with known (Bhagavat et al., 2018) or predicted (Wang et al., 2022; Sim et al., 2023) structures.

While the availability of large ligand structural libraries improves hit rates on pre-determined targets, the availability of large target structural libraries covering complete proteomes allows to perform near-complete screenings of hit and lead compounds for target selectivity. A major reason for candidate compound failure in drug discovery schemes is undesired or adverse effects, which is why characterization of absorption, distribution, metabolism, excretion and toxicity (ADMET) properties of candidate molecules at the earliest possible stage is relevant (Selick et al., 2002; Caldwell et al., 2009; Wu et al., 2020). Traditional ADMET prediction methods, such as quantitative structure activity relationship (QSAR) models, require costly and time-consuming data generation and are generally used relatively late in drug discovery programs. The increasing availability of data and resources enables ADMET predictions earlier in the process, with the use of machine learning tools to predict drug-target interactions, the blood-brain-barrier permeability of compounds, or toxic properties of drug candidates (reviewed in (Shaker et al., 2021)). The availability of predicted structures for complete proteomes should represent a paradigm shift in ADMET predictions, by affording approaches such as large-scale reverse docking, by which small molecules are simultaneously docked on many protein and cavity targets. This provides information on selectivity and thus potential off-target effects of the small molecules. For example, Wong et al. (2022) docked 319 compounds, of which 218 had antibacterial activity, on 296 essential *E. coli* proteins with structures predicted with AlphaFold2, finding unexpectedly promiscuous interactions and demonstrating the feasibility of the approach, albeit also highlighting the need to improve the performance of machine learning-based protein-ligand modeling methods.

Target-agnostic approaches have been applied as exploratory efforts to identify activities of interest prior to targeted drug discovery (Wang Y. et al., 2016). As such, metabolomics data can be integrated with data obtained by other omics techniques such as transcriptomics, proteomics or functional genomics with imaging-based or phenotypic screens (Kasap et al., 2014; Kurita et al., 2015; Bray et al., 2016; Subramanian et al., 2017; Earl et al., 2018; Setten et al., 2019; Ziegler et al., 2021). Eventually, as current criteria followed by drug approval agencies require the identification of molecular mechanisms, these exploratory approaches need to be followed up by biochemical, molecular and structural studies for a precise mechanistic characterizations of candidate drug activities.

## Perspectives and proposal

Significant constraints for the implementation of effective drug discovery programs in Latin America include relatively limited funding and failure to assemble coordinated transnational efforts. To date, scarce numbers of virtual screening projects in Latin America have led to the discovery of NP or NP-inspired compounds from isolation to proof-of-concept experimental activities of identified compounds (Fernandes et al., 2019; Rodrigues et al., 2019; Belgamo et al., 2020; Fernandez et al., 2020; Battini et al., 2021; Ferreira et al., 2021; Vargas et al., 2021; Valera-Vera et al., 2022; Adessi et al., 2023; Almeida et al., 2023; Araujo et al., 2023; Llanos et al., 2023; Peralta-Moreno et al., 2023). With the increasing availability and accessibility of advanced virtual screening tools that facilitate many stages in drug discovery pipelines (Daina and Zoete, 2019; Gentile et al., 2020; Ghislat et al., 2021; Singh et al., 2021; Arul Murugan et al., 2022; Blanes-Mira et al., 2022; Gorgulla et al., 2022; Muller et al., 2022; Sarkar et al., 2023; Thomas et al., 2023), which under conventional schemes are costly, labor intensive and time-consuming, a window of opportunity opens to change the tide towards NP-inspired drug discovery in less affluent economies.

Although less costly than conventional approaches, large next-generation virtual screening-centric drug discovery efforts based on NPs still require expertise and equipment for modern compound isolation and structural characterization, chemical synthetic and biosynthetic capabilities and, most importantly, ample computing power and connectivity. A further practical issue is the availability and cost of NPs and NP-inspired compounds for experimental validation of candidate molecules identified by virtual screening. The cost of NPs through conventional commercial channels can be relatively high, particularly for those with low yields in standard isolation procedures. As argued above, current technology enables early structural characterization of individual compounds, even as part of relatively complex mixtures, thus affording to bypass purification prior to structural characterization. In this scheme, NPs provide structures of interest, while experimental activity validation is performed with synthetic compounds that recapitulate NP structural features of pharmacological interest. This approach reduces the problem of yield, but does not totally solve the issue of cost per compound to be tested, particularly for those that may require difficult synthetic paths. New, more cost-effective synthetic strategies are expected to mitigate cost issues, as will entrusting non-profit institutions with on-demand synthesis of NP-inspired compounds for drug discovery. Together with building strong computational capabilities, this requires a concerted effort by individual teams, academic and industry organizations, transnational societies, institutional instances and public and private funding agencies, to design long-term, outcome-oriented plans coupled to commensurate multi-year funding schemes.
